# Antihepatitis B Virus Activity of a Protein-Enriched Fraction from Housefly (*Musca domestica*) in a Stable HBV-Producing Cell Line

**DOI:** 10.1155/2014/389560

**Published:** 2014-06-22

**Authors:** Xuemei Lu, Xiaobao Jin, Jie Wang, Fujiang Chu, Jiayong Zhu

**Affiliations:** ^1^Guangdong Provincial Key Laboratory of Pharmaceutical Bioactive Substances, Guangzhou Higher Education Mega Center, Guangdong Pharmaceutical University, 280 Wai Huan Dong Road, Guangzhou 510006, China; ^2^School of Basic Courses, Guangzhou Higher Education Mega Center, Guangdong Pharmaceutical University, 280 Wai Huan Dong Road, Guangzhou 510006, China

## Abstract

Hepatitis B virus (HBV) infection remains a major public health problem. Although several vaccines and therapeutic strategies are currently being implemented to combat HBV virus, effective antiviral therapy against HBV infection has not been fully developed. Alternative strategies and new drugs to combat this disease are urged. Insects and insect derivatives are a large and unexploited source of potentially useful compounds for modern medicine. In the present study, we investigated the first anti-HBV activity of a protein-enriched fraction (PE) from the larvae of the housefly (*Musca domestica*) in a stable HBV-producing cell line. HBsAg and HBeAg in the culture medium were measured by enzyme-linked immunosorbent assay. HBV-DNA was quantified by fluorescent quantification PCR. HBV core protein was assayed by immunofluorescent staining. Results indicate PE treatment inhibited both HBsAg, HBeAg secretion, and HBV-DNA replication. Furthermore, PE could also suppress HBV core protein expression. PE could be a potential candidate for the development of a novel and effective drug for the treatment of HBV infection.

## 1. Introduction

Hepatitis B virus (HBV) infection remains a major public health problem and causes transient and chronic hepatitis [1]. Approximately 3.5 hundred million individuals are infected with HBV worldwide, and approximately 1 million people die of HBV infection or related diseases each year [2]. Although several vaccines and therapeutic strategies are currently being implemented to combat HBV virus, no effective antiviral therapy against HBV infection has yet been fully developed [3]. The side effects of current pharmacological agents and development of drug-resistance in HBV are emerging clinical problems [4, 5]. Thus, alternative strategies and new drugs to combat this disease are urged.

Insects and insect derivatives are a large and unexploited source of potentially useful compounds for modern medicine [6]. Housefly (*Musca domestica*) can thrive without causing infection in the most unfavorable surroundings, largely due to its antimicrobial and immunoactive substances in the immune system [7].* Musca domestica *larvae are traditionally used as therapeutic agents for decubital necrosis, malnutritional stagnation, and ecthyma since the Ming/Qing Dynasty (1368 Anno Domini) up to now in China [8]. Recently, the effects of antioxidant [9], antibacterial [10], antitumor activities [11], and immunomodulatory functions [12] of the protein-enriched extracts of housefly larvae have been reported. Additionally, the protein-enriched extracts of housefly larvae have been reported to possess potential hepatoprotective effect [13] and antiviral activity against pseudorabies virus [14] and influenza virus [7]. However, it is unclear whether the protein-enriched extracts of housefly larvae have potent antiviral activity against hepatitis B virus.

In the present study, HepG2.2.15 cell, which has been stably transformed with two copies of the HBV genome into human hepatoblastoma cell line HepG2, was used as* in vitro* model to evaluate the anti-HBV effect of protein-enriched fraction (PE) from* Musca domestica* larvae. HBsAg and HBeAg in the culture medium were measured by enzyme-linked immunosorbent assay. HBV-DNA was quantified by using fluorescent quantification PCR. HBV core protein in HepG2.2.15 cell was assayed by immunofluorescent staining and scanned by confocal microscope.

## 2. Methods

### 2.1. Preparation of Protein-Enriched Fraction from* Musca domestica* Larvae

*Musca domestica* was supplied by Guangdong Provincial Center for Disease Control and Prevention (CDC), China. The protein-enriched fraction of* Musca domestica* larva was prepared from* Musca domestica* larva as described before [12]. First, the third-instar larvae were collected, washed with distilled water, and frozen at −20°C. Then, the larvae were weighted and homogenized with a homogenizer (4 pulses, 5 sec at 3500 rpm) on ice. The homogenate was filtered with a layer of 64 mm nylon mesh and then incubated at 4°C for 60 min. After removing the insoluble material, the solution was centrifuged at 1300 g for 10 min at 4°C. The supernatant was concentrated and lyophilized. The protein concentration was determined using the bicinchoninic acid (BCA) protein assay kit according to the manufacturer's instructions. The mass of lyophilized powder was weighed and the content of protein was calculated. The protein fractions were stored at −80°C.

### 2.2. Cell Culture

HepG2.2.15 cells were grown in complete DMEM (Gibco-BRL, CA) containing 10% FBS (Hyclone, Thermo Fisher, PA), 100 units/mL penicillin, 100 mg/mL streptomycin, and 380 micro·g/mL G418 antibiotic (Sigma, MO) in a humidified incubator with 5% CO_2_ at 37°C.

### 2.3. Cytotoxicity Measurement

Cytotoxicity was assessed by the MTT assay as previously described [15]. Briefly, HepG2.2.15 cells were cultured in 96-well plates (2 × 10^4^/well) for 9 days with various concentrations of PE. The cells with media alone were used as controls. The culture medium was replaced every 3 days. At the end of the culture, MTT solution (5 g/L) was added, and incubated another 4 h at 37°C. Finally, DMSO was added to solubilize the formazan, and the optical density (OD) values were read at 450 nm. The survival ratio (%) was calculated using

(1) $$\begin{array}{c} \text{Survival}\;\text{rate}\;\left(\%\right)=\left(\frac{{\text{OD}}_{450}\;\text{of}\;\text{experimental}\;\text{group}}{{\text{OD}}_{450}\;\text{of}\;\text{negative}\;\text{control}}\right)\times 100\%. \end{array}$$

### 2.4. Antiviral Assays

Antiviral assays were conducted as previously described [15, 16]. Briefly, the HepG2.2.15 cells were seeded in 24-well plates (2 × 10^5^/well) and incubated to reach confluence. Then the culture medium was replaced by fresh medium with different concentrations of PE or ADV (adefovir, positive control) for 3, 6, and 9 days. The cells with media alone were used as controls. The culture medium was replaced every 3 days. The supernatants were collected for HBV antigens and extracellular HBV-DNA assays, respectively. The cells were collected for the intracellular HBV-DNA assays on the 9th day.

### 2.5. Measurement of Secreted HBsAg and HBeAg

HBsAg and HBeAg in the culture medium were measured by enzyme-linked immunosorbent assay (ELISA) using HBsAg and HBeAg diagnostic kit (Shanghai Kehua Biotech Co., Ltd., China) according to the manufacturer's recommendations. The OD values at 450/630 nm were read. The inhibitory rates were calculated using

(2) $$\begin{array}{c} \text{Inhibitory}\;\text{rate}\;\left(\%\right)=\left\{\frac{\left({\text{OD}}_{\text{control}}-{\text{OD}}_{\text{PE}}\right)}{{\text{OD}}_{\text{control}}}\right\}\times 100\%. \end{array}$$

### 2.6. HBV-DNA Quantification by Fluorescent Quantification PCR

HBV-DNA was quantified using fluorescent quantification PCR [17]. Viral DNA was extracted from the culture medium and cells, and then proper aliquots were used for the fluorescent quantification PCR. HBV fluorescent quantitative PCR diagnostic kit (Da-An Gene Co., Ltd., China) was used to determine the load of HBV viral according to the manufacturer's protocol. The PCR reaction was carried out as follows: initially denaturation at 93°C for 2 min followed by 10 cycles of 93°C for 45 s and 55°C for 60 s and 30 cycles of 93°C for 30 s and 55°C for 45 s. HBV-DNA was quantified using a standard curve. The inhibition ratio (%) was calculated using

(3) $$\begin{array}{c} \text{Inhibitory}\;\text{rate}\;\left(\%\right)=\left\{\frac{(\text{HBV-DNA}\;{\text{concentration}}_{\text{control}}\;-\;\;\text{HBV-DNA}\;{\text{concentration}}_{\text{PE}})}{\text{HBV-DNA}\;{\text{concentration}}_{\text{control}}}\right\}\times 100\%. \end{array}$$

### 2.7. Immunofluorescent Staining and Confocal Microscope Analysis for HBV Core Protein

A single HepG2.2.15 cell suspension was seeded on coverslip in six-well plate (2 × 10^5^/well) overnight. Cells were treated with different concentrations of PE or ADV (adefovir, positive control) for 9 days and fixed with 4% paraformaldehyde and permeabilized with 0.1% (vol/vol) Triton X-100 for 30 min. The primary antibody is rabbit polyclonal IgG anti-human HBcAg (1 : 200; Abcam, England) and the secondary antibody is Alexa 488 conjugated goat anti-rabbit IgG (1 : 200; Jackson ImmunoResearch, USA). The chromosome was stained with 4′,6-diamidino-2-phenylindole (DAPI, Vector, CA) for nuclear indication. Images were captured using a confocal laser scanning microscope (TCS-NT, Leica Microsystems, Heidelberg, Germany).

### 2.8. Statistical Analysis

All data were expressed as the mean ± standard error of the mean (SEM). Differences between mean values were analyzed using one-way ANOVA. All statistical analyses were performed by SPSS (version 13.0 for Windows) statistical software. Each measurement was performed at least in triplicate.

## 3. Results

### 3.1. The Protein Content and Cytotoxicity Measurement

**Figure 1:**
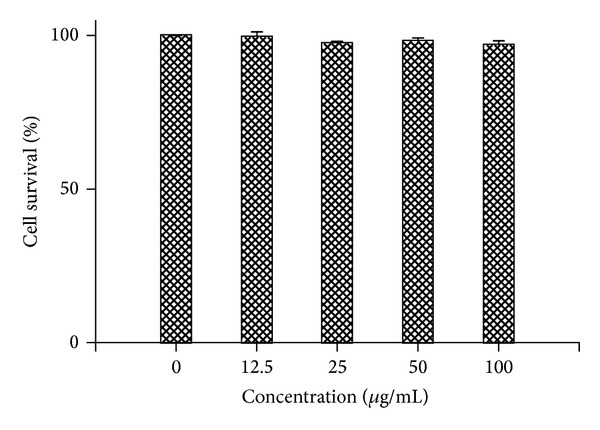
HepG2.2.15 cells were cultured in the presence of PE at various concentrations for 9 days and the cell survival rates were measured by MTT method. Data represent the mean ± SEM (*n* = 4).

The protein content of PE was about 99.18 ± 0.27%. The cell survival rate of the HepG2.2.15 cell in the presence of various concentrations of PE was tested. As shown in Figure 1, no obvious cytotoxicity was observed for PE, even at concentration of 100 micro*·*g/mL for 9 days.

### 3.2. Anti-HBV Antigens Secretion Activity of PE

**Figure 2:**
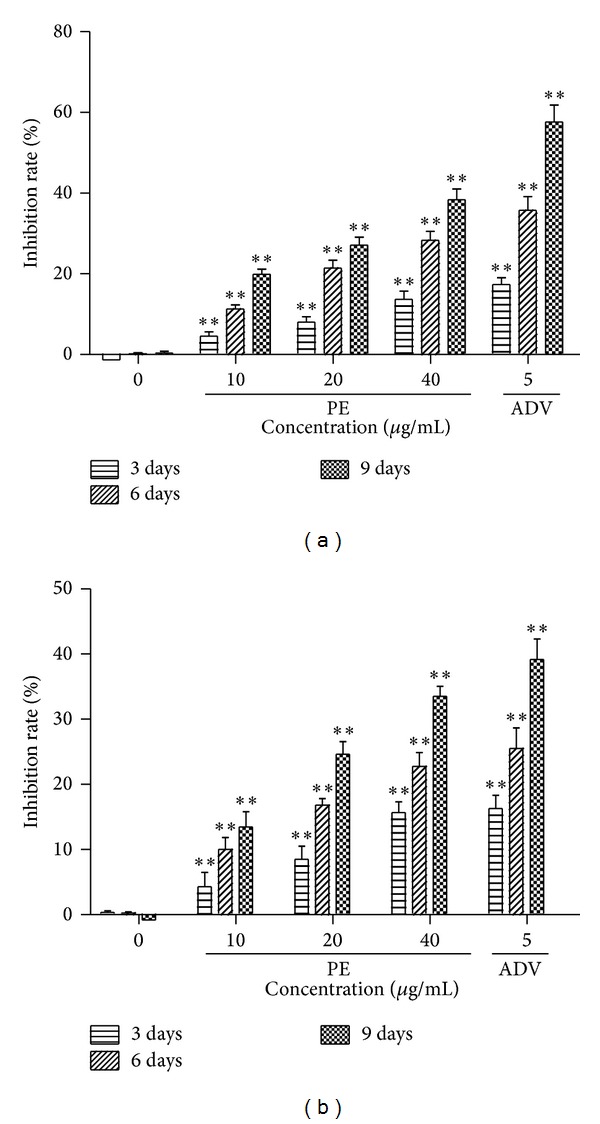
PE treatment suppressed HBsAg and HBeAg secretion. HepG2.2.15 cells were treated with or without drug at the indicated doses for 3, 6, and 9 days and hepatitis B surface antigen (HBsAg; (a)) and hepatitis B e-antigen (HBeAg; (b)) levels were determined by ELISA. Data represent the mean ± SEM of three experiments. **P < 0.01 as compared with the no-drug control group.

Based on the results of cytotoxicity, we selected three treatment concentrations of PE with the highest dose at 40 micro·g/mL to evaluate its antiviral effect. The levels of HBsAg and HBeAg in the supernatant of HepG2.2.15 cell culture system were measured by ELISA using HBsAg and HBeAg diagnostic kit. Treatment of HepG2.2.15 cells with different concentrations of PE at different times resulted in a significant reduction of HBsAg and HBeAg secretion (Figure 2) in a dose-dependent manner.

### 3.3. Effect of PE on HBV-DNA Replication

**Figure 3:**
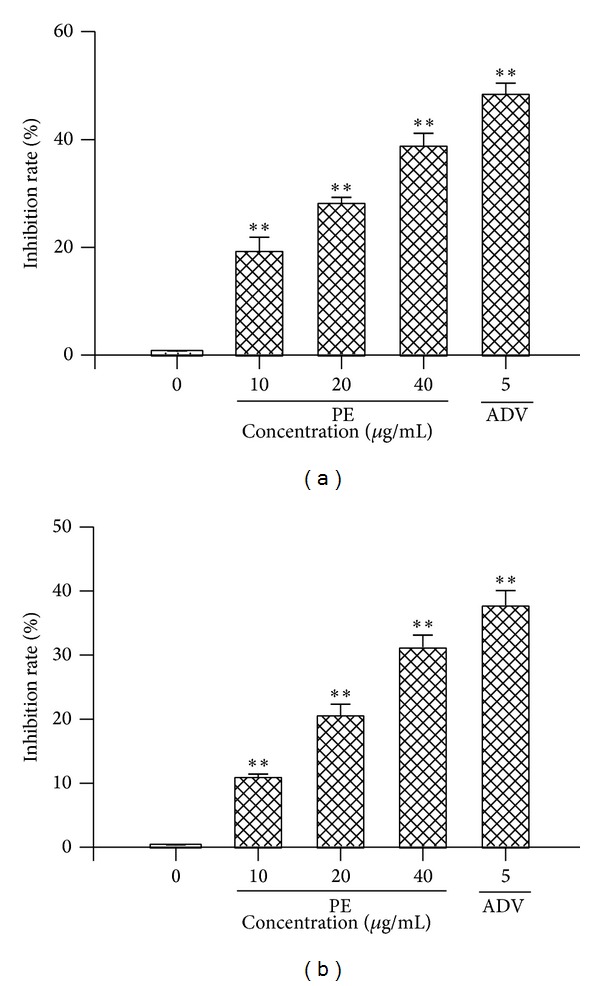
Inhibitory effect of PE on HBV-DNA level in HepG2.2.15 cells. HepG2.2.15 cells were treated with or without drug at the indicated doses for 9 days. HBV-DNA levels were measured by real-time quantitative PCR. Data represent the mean ± SEM of three experiments. **P < 0.01 as compared with the no-drug control group. (a) Extracellular and (b) intracellular.

The results of fluorescent quantification PCR revealed that treatment with various concentrations of PE for 9 days result in the reduction of intracellular and extracellular HBV-DNA levels, as compared with a control group (*P* < 0.05). As the figure shows (Figure 3), the mean inhibition percentage of viral DNA levels with PE at the dosages of 10 micro·g/mL, 20 micro*·*g/mL, and 40 micro*·*g/mL were 19.48%, 28.39%, and 39.02% extracellularly and 13.15%, 20.39%, and 31.24% intracellularly, respectively.

### 3.4. Inhibit the Expression of HBV Core Protein in HepG2.2.15 Cells by PE Treatment

**Figure 4:**
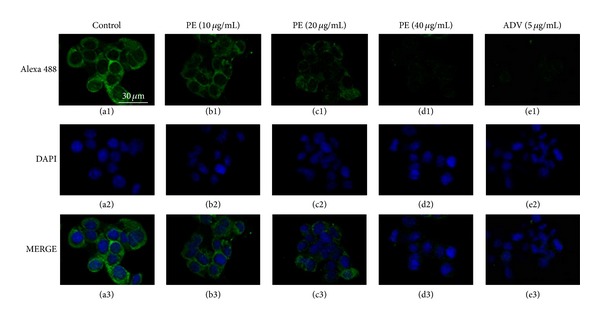
Analysis of HBV core protein in HepG2.2.15 cells treated with PE. Cells were treated with or without drug at the indicated doses for 9 days and stained by immunofluorescent staining. Cells were scanned for subcellular distribution of HBV core protein (Green) by confocal microscope. Blue nuclear stained with DAPI. Calibration bar = 30 *μ*m for all images.

HepG2.2.15 cells were treated with PE at the concentrations of 10 micro*·*g/mL, 20 micro*·*g/mL, and 40 micro*·*g/mL for 9 days. HBV core protein was assayed by immunofluorescent staining and scanned by confocal microscope. The results (Figure 4) showed that the signal of core protein is obviously decreased in the cytoplasm of HepG2.2.15 cells treated with PE in a dose-dependent manner when compared with the control cells.

## 4. Discussion

There are two arms of therapy to manage HBV infection, either by direct antiviral therapy to inhibit replication of HBV or by indirect immunomodulatory therapy to enhance cellular immunity to combat this disease [18]. Direct antiviral therapy with nucleoside analogues (e.g., lamivudine entecavir, dipivoxil,adefovir, and tenofovir) could efficiently control hepatitis B, but drug resistant mutant could be developed progressively after initiation of therapy [19]. Indirect immunomodulatory therapy with interferon alpha (IFN *α*) had low efficiency with many limitations [20]. Moreover, major side effects and high costs of these agents strengthened the need for new anti-HBV agents.

Hundreds of traditional Chinese medicines (TCMs) have been used for hepatitis B treatment [21–23]. For example, typical strategies of TCM treatment in hepatitis B are (1) to decrease the load of HBV viral; (2) to improve liver function; (3) to ameliorate liver inflammation; (4) to improve immune function; (5) to regulate lipid metabolism; and (6) to ameliorate hepatic fibrosis [21, 22]. The protein-enriched fraction extract from traditional Chinese medicines, housefly larvae, has been found to possess potential hepatoprotective effect [13], immunomodulatory function [12], inflammation regulation effect [24], and antiviral activity against pseudorabies virus [14] and influenza virus [7]. However, the anti-HBV active of the protein-enriched extracts of housefly larvae, until now, has not been studied. In the present study, our results, for the first time, clearly demonstrated that the protein-enriched extracts of housefly larvae could inhibit HBV replication in an HBV-producing cell line. We speculate that PE treatment may benefit the liver by protecting the liver, inhibiting inflammation, inhibiting HBV replication, and enhancing cellular immunity. So the protein-enriched extracts of housefly larvae could be an excellent candidate for the development of potential HBV therapeutic agent.

Before identifying the effective dose which interfered with HBV replication and viral protein production, it is critical to use concentrations that are not overtly cytotoxic, since any impairment to cell functions would affect virus replication. Our results showed that the administration of even 100 micro*·*g/mL of PE for 9 days did not induce cell death, indicating PE achieved the virus inhibiting effect without cytotoxicity on HepG2.2.15 cell lines. The HepG2.2.15 cell line was obtained by stable transfection of the human HepG2 hepatoma cell line with a plasmid containing two copies of the HBV genome (subtype ayw) [25]. The cell line could support the full replication cycle HBV and incomplete double- and single-stranded forms of the HBV genome. Therefore, the cell line is an appropriate* in vitro* model for identifying the secretion of HBV particles as well as the molecular events in intracellular viral replication cycle. PE has shown its inhibitory effect on HBV proliferation* in vitro* by decreasing the production of HBsAg and HBeAg, which are two predominant indications of HBV infection and can be detected in the supernatant. Consistent with the inhibitory effects on productions of HBsAg and HBeAg (Figure 2), significant depression in intracellular and extracellular HBV-DNA levels was observed (Figure 3). Moreover, the results of immunofluorescent staining showed that PE obviously decreased the level of cytoplasmic HBV core protein (Figure 4). These results suggested that the protein-enriched extracts of housefly larvae functioned to inhibit the HBV replication, viral protein production, and secretion.

In conclusion, we demonstrated for the first time that protein-enriched extracts of housefly larvae efficiently inhibited the expression of HBV virus proteins and the HBV-DNA replication in HepG2.2.15 cell line* in vitro*. PE has potential anti-HBV activities, which support its use in traditional Chinese medicines, and it can be further studied for the drugs development to treat HBV infection patients.
